# Task-specific oscillatory synchronization of prefrontal cortex, nucleus reuniens, and hippocampus during working memory

**DOI:** 10.1016/j.isci.2023.107532

**Published:** 2023-08-03

**Authors:** Johanne Gertrude de Mooij-van Malsen, Niels Röhrdanz, Anna-Sophia Buschhoff, Thomas Schiffelholz, Torfi Sigurdsson, Peer Wulff

**Affiliations:** 1 Institute of Physiology, Christian-Albrechts-University Kiel, Kiel, Germany; 2 Center of Integrative Psychiatry, University Medical Center Schleswig-Holstein, Kiel, Germany; 3 Institute of Neurophysiology, Neuroscience Center, Goethe University, Frankfurt, Germany

**Keywords:** Neuroscience, Behavioral neuroscience, Cognitive neuroscience

## Abstract

Working memory requires maintenance of and executive control over task-relevant information on a timescale of seconds. Spatial working memory depends on interactions between hippocampus, for the representation of space, and prefrontal cortex, for executive control. A monosynaptic hippocampal projection to the prefrontal cortex has been proposed to serve this interaction. However, connectivity and inactivation experiments indicate a critical role of the nucleus reuniens in hippocampal-prefrontal communication. We have investigated the dynamics of oscillatory coherence throughout the prefrontal-hippocampal-reuniens network in a touchscreen-based working memory task. We found that coherence at distinct frequencies evolved depending on phase and difficulty of the task. During choice, the reuniens did not participate in enhanced prefrontal-hippocampal theta but in gamma coherence. Strikingly, the reuniens was strongly embedded in performance-related increases in beta coherence, suggesting the execution of top-down control. In addition, we show that during working memory maintenance the prefrontal-hippocampal-reuniens network displays performance-related delay activity.

## Highlights

• A touchscreen-based working memory task allows temporal separation of memory phases
• Oscillatory synchronization of mPFC, nRe, and CA1 changes with phase and difficulty
• During choice, the nRe participates in beta and gamma but not theta synchronization
• All structures engage in performance-related delay activity during memory maintenance

## Graphical abstract

**Figure:**
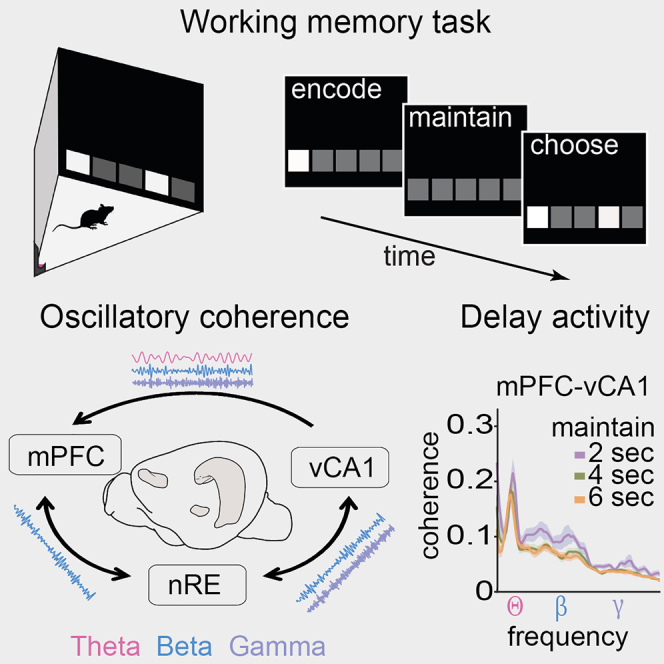

## Introduction

Working memory (WM) can be defined as a short-lasting online memory buffer which holds information that is relevant to an ongoing task and is thought to rely on two parallel processes: online maintenance of information and its executive control.^1^^,^^2^ WM is critical not only for high-performance intellectual abilities but essential for accomplishing everyday life. Accordingly, impairment of WM as occurs with chronic stress or as core cognitive symptom in psychiatric disorders such as schizophrenia or depression represents a major debilitating factor.^3^^,^^4^^,^^5^^,^^6^^,^^7^ In mammals WM critically depends on the prefrontal cortex (PFC). However, this brain region does not act alone but rather relies on functional connectivity with other brain regions for the execution of WM.^3^ In spatial WM - one of the best studied forms of WM - inactivation studies have shown that both the PFC and the hippocampus (HC) and ultimately the functional interaction between these brain regions are required.^8^^,^^9^^,^^10^^,^^11^^,^^12^ Consistent with these findings, recordings of neuronal activity during WM tasks have revealed enhanced oscillatory coherence of the HC and medial PFC (mPFC) at different frequency bands, which in turn correlates with task performance.^13^^,^^14^^,^^15^^,^^16^^,^^17^^,^^18^^,^^19^ Along these lines it has been shown that diseases and interventions that compromise hippocampal-prefrontal connectivity and coherence also impair WM performance.^17^^,^^18^^,^^19^^,^^20^^,^^21^

Regarding the anatomical substrates of HC-PFC interactions, presumably the best studied connection is a direct, mono-synaptic projection from the ventral hippocampal CA1 region (vCA1) to the medial PFC.^22^ The direction of this projection is in agreement with the direction of information flow during WM-related increases in theta coherence between these brain regions as computed from neuronal recordings^13^^,^^14^^,^^15^^,^^16^^,^^17^ (but see^18^). However, in addition to this mono-synaptic projection, the HC and PFC are also connected via poly-synaptic pathways.^23^^,^^24^ The most prominent of these is relayed through the nucleus reuniens of the thalamus - the largest of the midline thalamic nuclei (nRE; for review see^23^^,^^24^^,^^25^). Here, projection neurons in the deep layers of mPFC directly synapse onto nRE neurons which project to stratum lacunosum-moleculare of the vCA1 region to synapse onto distal dendrites of excitatory and inhibitory neurons providing a strong thalamic input to the HC.^26^^,^^27^^,^^28^^,^^29^^,^^30^^,^^31^^,^^32^ However, the nRE also receives dense afferents from vCA1/Subiculum and sends strong efferents to superficial and deep layers of the pre- and infralimbic cortices of the mPFC.^28^^,^^33^^,^^34^ The nRE has thus been suggested to serve as a central hub in bidirectional HC-mPFC communication, a notion that is supported by functional studies that showed reduced mPFC-CA1 synchrony after nRE inactivation and behavioral studies which showed that nRE inactivation affects especially those behaviors, which depend on prefrontal-hippocampal communication including WM.^35^^,^^36^^,^^37^^,^^38^^,^^39^^,^^40^^,^^41^^,^^42^^,^^43^^,^^44^

In the current study we have investigated the dynamics of interregional communication in the mPFC-nRE-vCA1 network by analyzing the coherence of local field potentials (LFPs) across the three nodes at different stages of a touch-screen based WM task. We found that communication within the network occurs at distinct frequencies depending on the immediate cognitive demand at hand and its difficulty. In particular we report performance related increases in mPFC-nRE-vCA1 beta coherence during decision periods as well as performance-related oscillatory delay activity during WM maintenance.

## Results

### Implementation of a touch-screen-based working memory task

**Figure 1:**
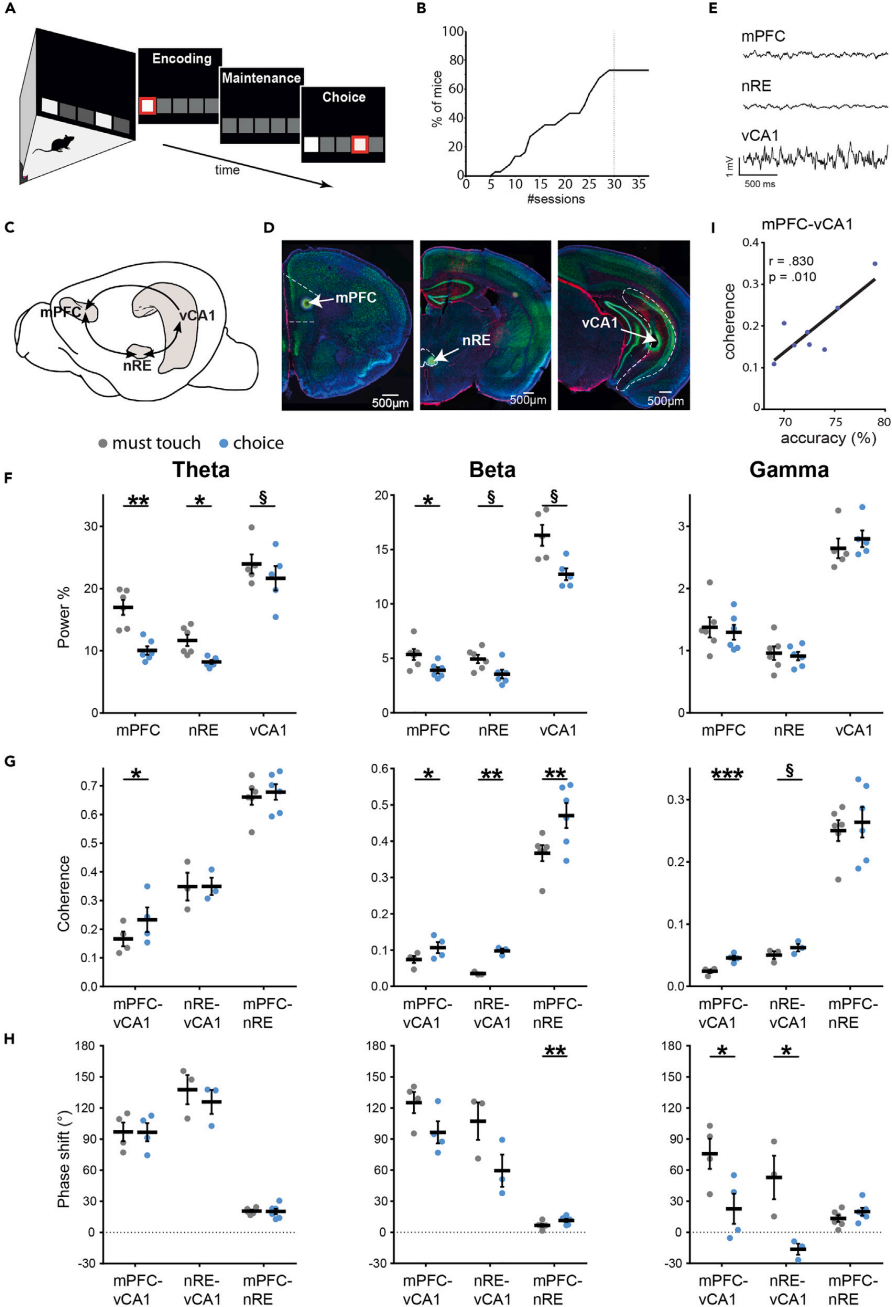
Coherence in the vCA1-mPFC-nRE network increases at theta, beta and gamma frequency during working memory-based choice (A) Cartoon of the behavioral protocol illustrating the configuration of illuminated fields displayed sequentially during one DNMTS trial within the touch-screen-chamber. The respective field that has to be touched is indicated by a red frame. Sample and test location are randomly altered each trial. (B) The percentage of animals reaching criterion increased with successive training sessions. Mice that had not learned the task by 30 sessions (one 30-min session per day) did not improve with additional sessions and were excluded. (C) Illustration of anatomical positions of mPFC, nRE, and vCA1 in the mouse brain. Arrows indicate directionality of anatomical connections. (D) After recordings, small electrolytic lesions were induced to mark electrode positions. These were identified (arrows) in coronal sections immuno-labeled for NeuN (green), GFAP (red). (E) Examples of 2 s raw LFP traces recorded in the mPFC, the nRE, and the vCA1 during choice. (F) Percentage of total power within the theta (left), beta (middle) and gamma (right) range for the WM-choice and the non-WM must-touch-task. Theta and beta power decreased in all three structures during WM-choice, whereas gamma power did not change. (G) Coherence at theta frequency (left) between vCA1 and mPFC is significantly higher during WM choice than during non-WM must-touch, however theta coherence between nRE and the other two regions does not change. Coherence within the beta range (middle) increased during the choice phase in all connections compared with the non-WM must-touch-task. Within the selected gamma range (right), coherence increased between mPFC and vCA1, and marginally between the nRE and vCA1. (H) Phase shift analysis showed no significant differences between choice and must-touch task at theta frequency (left). Significant changes in phase were found during choice between nRE and mPFC in the beta range (middle) as well as between both, mPFC and nRE on the one hand and vCA1 on the other for gamma oscillations (right). Positive values indicate that the first structure is shifted in time against the second structure. (I) mPFC-vCA1 theta coherence is correlated with choice accuracy. Abbreviations: mPFC, medial prefrontal cortex; nRE, nucleus reuniens; vCA1, ventral CA1; Θ, Theta. Data are shown as mean ± SEM. ∗p ≤ 0.05, ∗∗p ≤ 0.01, ∗∗∗p ≤ 0.001, § 0.05 < p < 0.1 (trend).

WM is commonly analyzed in delayed non-match to sample (DNMTS) paradigms, where a subject after a delay period has to choose the stimulus that has not previously been presented.^45^ In rodents such DNMTS tasks are predominantly implemented in T-mazes, which require little training and achieve high performance levels even with prolonged delay intervals. Alternatively, more demanding two-lever DNMTS boxes have been used.^46^ However, in both behavioral paradigms the animal only has a left and a right option in the choice run and could thus generate an action plan already in the sample run, making it difficult to gauge the impact and temporal structure of WM, in particular as confounding motor strategies (e.g., positioning head or body toward the opposite side after the sample run) may be applied (see also^18^^,^^47^^,^^48^). To overcome these difficulties, we have implemented a DNMTS protocol in automated mouse touch screen chambers^47^ (Figure 1A), which is more similar to WM tests in primates and puts particular strain on hippocampal-prefrontal communication.^49^^,^^50^ In this task mice have to respond by touch to an illuminated sample at a random (five options) screen location during a sample phase (encoding). This sample touch is rewarded at the opposite side of the conditioning chamber. After a delay interval of initially 2 s (maintenance), the sample and a novel random test location are illuminated and the animal needs to respond to the novel location (choice), which is rewarded (Figure 1A). As the animals have to visit the opposite side of the chamber after the sample touch and the test location can then be illuminated on either side of the sample location, the risk of post-sample action planning and confounding motor strategies is reduced. Accordingly, the correct choice has to be made based on WM of the sample location, which in turn permits a more reliable temporal separation of WM phases. Training the animals with a maintenance period of 2 s with one training session of 30 min per day we found that 76% of mice were capable of learning the task to criterion (>70% accuracy for three consecutive days) in a maximum of 30 sessions (average: 20 ± 1.7 sessions; Figure 1B).

To investigate communication in the mPFC-nRE-vCA1 network (Figure 1C) during the task, mice performing stably above threshold were implanted with electrodes bilaterally in the mPFC (prelimbic cortex), the nRe and the vCA1 (near stratum lacunosum-moleculare) for recordings of LFPs (Figures 1C–1E and S1). After recovery, mice were initially recorded in a simple response task, in which they had to touch a single illuminated location upon its first appearance to receive a reward (non-WM: must-touch task). Subsequently they were recorded in the full WM-task. The time line of the entire experimental protocol and individual recording trials in the must-touch and WM-task are illustrated in Figure S2. To probe for WM-related activity across the mPFC-nRE-vCA1 network we first compared power and phase coherence of LFPs in the last 2 s of the choice phase of the WM-task against the last 2 s in the must-touch task. The only difference between these two settings was that in the WM-task the mouse had to choose between two illuminated locations based on WM, whereas in the must-touch task it had to simply touch a single illuminated location without requirement for WM (Figure S2).

Spectral analysis indicated differences in power, coherence and phase relations between WM and non-WM tasks in specific frequency bands (Figure S3). Accordingly, for subsequent analysis we focused on the core frequencies of 7–9 Hz, 19–30 Hz and 40–50 Hz, which lay in the theta, beta and gamma frequency range, respectively, to analyze differences between the tasks.

### Theta coherence increases with WM between vCA1 and mPFC but does not involve the nRE

It has been reported that theta oscillations play a prominent role in hippocampal-prefrontal communication during choice phases in T-maze WM tasks.^14^^,^^15^^,^^17^^,^^51^ We thus analyzed whether this frequency band was also relevant in our touch screen-based paradigm. In accordance with previous reports we found prominent oscillatory activity in the theta range (peak frequency of about 8 Hz) in vCA1 and mPFC^14^^,^^15^^,^^17^^,^^51^ but also the nRE (Figures 1F and S3A). Since running speed is known to influence the magnitude of hippocampal theta oscillations, we tested whether running affected vCA1 theta power in our touch-screen paradigm by correlating speed and theta power of individual animals in the must touch and DNMTS task on a trial to trial basis. We found no correlation (must touch: r = 0.110, p = 0.258, DNMTS: r = 0.099, p = 0.311, n = 107 trials each, 6 mice), suggesting that running speed had no major effect on theta power in our task. In addition, running speed did not differ in must touch and DNMTS tasks (t(4) = -1.998, p = 0.116). When we compared theta power between non-WM must touch and WM-choice phases, we found that power was lower during WM in all three regions, suggesting that local activity at theta frequency was stronger in the must-touch task (Figure 1F; mPFC t(5) = 6.522, p = 0.001; nRE t(5) = 3.361, p = 0.020; vCA1 t(4) = 2.484, p = 0.068). However, when we analyzed inter-regional coherence as a measure of information flow between regions,^52^ we found that coherence at theta frequency was specifically enhanced between mPFC and vCA1 during WM choice (mPFC-vCA1 t(3) = 3.249, p = 0.048); nRE- CA1 t(2) = 0.024, p = 0.98; mPFC-nRE t(5) = 0.822, p = 0.45) (Figures 1G and S3B), consistent with previous data from T-maze experiments.^14^^,^^15^^,^^17^^,^^18^^,^^24^^,^^51^ In contrast, theta coherence between mPFC and nRE or nRE and vCA1 did not differ significantly between must-touch task and WM choice phase (Figures 1G and S3B). Theta coherence between vCA1 and mPFC correlated significantly with choice accuracy during WM (r(6) = 0.83, p = 0.01; Figure 1I), suggesting that mPFC-vCA1 theta coherence is linked to WM performance not only in T-maze^14^^,^^15^^,^^17^ (but see^53^) but also in our touch screen tasks.

Phase shift analysis suggested that mPFC theta was shifted against vCA1 theta by about 90° (Figures 1H and S3C; see also^15^^,^^16^^,^^24^). Similarly, the nRE was shifted against vCA1 (Figures 1H and S3).

Together these data show that theta oscillations are prominent in vCA1, mPFC and nRE with high coherence between the regions. During the choice phase of WM, we find a specific and performance related increase in theta coherence between vCA1 and mPFC. The nRE does not participate in the enhanced theta coherence, suggesting that during choice, theta-related information flow between vCA1 and mPFC does not involve the nRE.

### Beta and gamma coherence in the mPFC-nRE-vCA1 network increase during WM choice

Similar to theta oscillations, also power in the beta range was lower in all three brain regions during the choice phase of WM than during the non-WM must-touch-task, although this reduction was significant only for the mPFC (Figures 1F and S3A; mPFC t(5) = 2.952, p = 0.032; nRE t(5) = 2.047, p = 0.096; vCA1 t(4) = 2.717 p = 0.053). Remarkably, coherence between all structures in the network was significantly increased in this frequency range (Figures 1G and S3B; mPFC-vCA1 t(3) = 3.304, p = 0.046; nRE-vCA1 t(2) = 11.980, p = 0.007; nRE-mPFC t(5) = 4.339, p = 0.007). Similar increases in beta coherence have previously been reported for simultaneous recordings from mPFC and mediodorsal thalamus (MD) as well as mPFC and dorsal CA1 during WM.^53^^,^^54^^,^^55^ The phase of beta oscillations in mPFC and nRE was shifted against vCA1 and nRE phase was shifted against mPFC with a slight increase in shift during choice compared to must-touch (nRE-mPFC t(10) = 2.253, p = 0.048; unpaired t-test) (Figures 1H and S3C).

In the gamma range, power was not different between non-WM must-touch and WM-choice tasks in any of the three structures (Figures 1F and S3A). However, gamma coherence showed highly significant increases during choice compared to must-touch between mPFC and vCA1 (Figures 1G and S3B; mPFC-vCA1 t(3) = 13.760, p = 0.0008), similar to changes reported from T-maze DNMTS tasks.^18^^,^^19^ In addition we found marginal increases in gamma coherence between nRE and vCA1 (Figure 1G; nRE-vCA1 t(2) = 3.272, p = 0.082). Interestingly, these increases in coherence were accompanied by changes in phase relations with significant reductions in phase difference between mPFC and vCA1 (vCA1 leading, t(6) = 2.579, p = 0.042), and even a change from positive during must-touch to negative during choice between nRE and vCA1(t(4) = 3.206, p = 0.033) (Figures 1H and S3C).

Together, these data show enhanced coherence within the entire mPFC-nRE-vCA1 network during the choice phase of WM at beta frequency, and between mPFC and vCA1 as well as nRE and vCA1 in the gamma range with a change in phase relations between nRE and vCA1 as well as mPFC and vCA1 at this frequency. These findings indicate that the nRE participates in hippocampal-prefrontal communication at beta and gamma frequency during the choice phase of WM.

### Phase coherence between nRE and vCA1 during choice increases with task difficulty and correlates with WM performance

**Figure 2:**
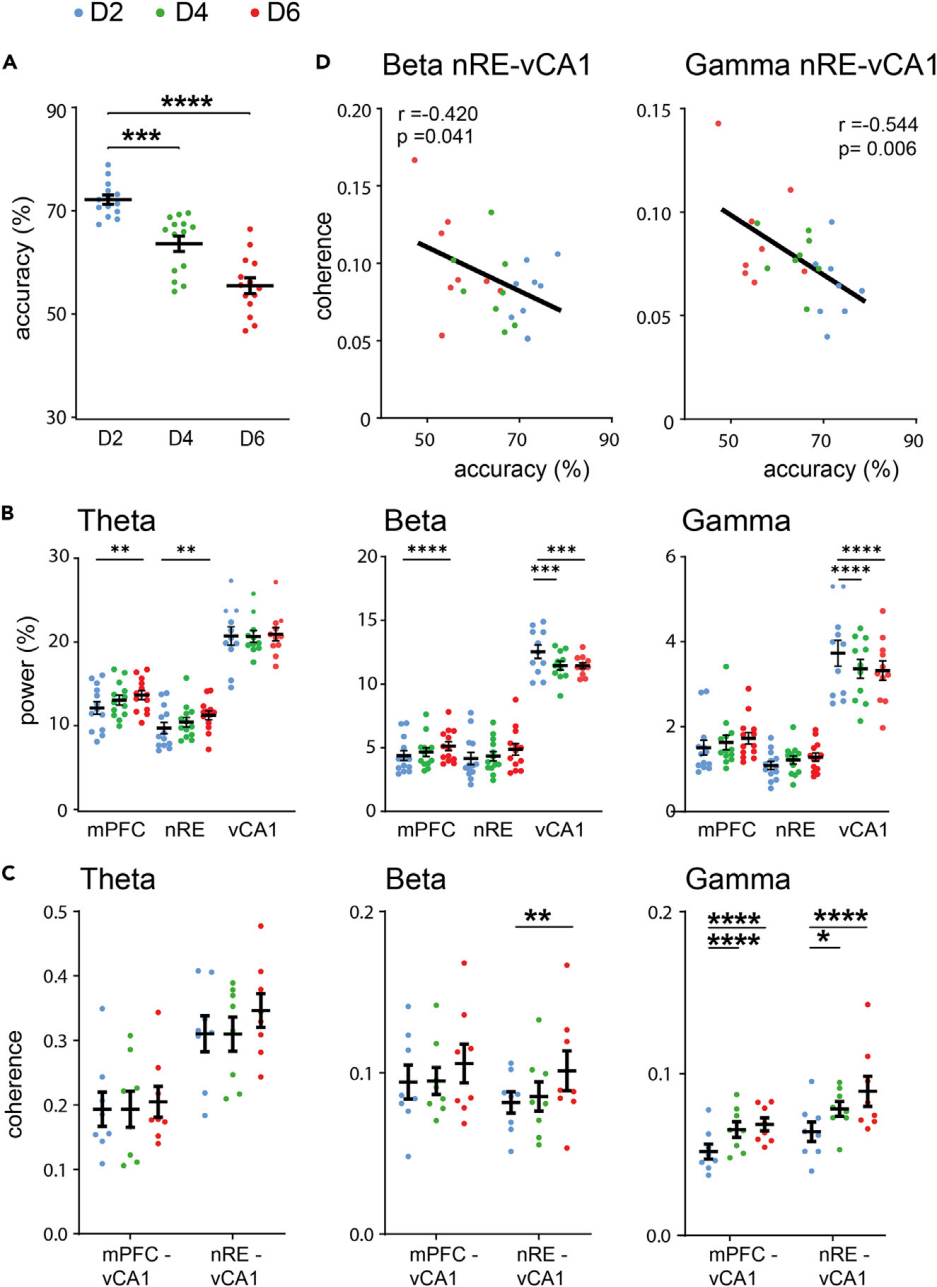
Oscillatory coherence during the choice phase of working memory increases with task difficulty and correlates with performance (A) Choice accuracy declined significantly with extended WM delay periods. Average accuracy is shown per animal during choice phases after a 2 s (D2; blue), 4 s (D4; green) or 6 s (D6; red) delay. (B) Changes in theta (left), beta (middle) and gamma (right) power (percentage of total power) in mPFC, nRE and vCA1 during the retrieval phase after 2, 4 and 6 s WM delay intervals. (C) Increased delay durations caused an increase in coherence between the nRE and vCA1 in both the beta and gamma range, as well as for mPFC-vCA1 within the gamma range during choice. (D) Coherence levels during choice between nRE and vCA1 within the beta and gamma range are negatively correlated with accuracy levels during WM. Abbreviations: mPFC, medial prefrontal cortex; nRE, nucleus reuniens; vCA1, ventral CA1. Data are shown as mean ± SEM.∗p ≤ 0.05, ∗∗p ≤ 0.01, ∗∗∗p ≤ 0.001, ∗∗∗∗p ≤ 0.0001.

To probe whether increased task demands would be reflected by changes in network activity, we extended the delay period from 2 s to 4 and 6 s (D2, D4 and D6). This required the animals to hold in mind the sample position for a prolonged period (maintenance) and thus increased task difficulty.^19^^,^^56^ As expected, choice accuracy decreased significantly with delay duration (Figure 2A; D2: 72.3 ± 0.88%; D4: 63.8 ± 1.48%; D6: 55.7 ± 1.52%; repeated measures Anova (rma): F(1.779, 23) = 50,63, p < 0.0001; post-hoc Dunnett (ph): D2-D4 p = 0.001; D2-D6 p < 0.0001).

With longer delay durations theta power during the choice phase increased significantly in the mPFC (rma: F(2, 24) = 14.63, p < 0.0001; ph: D2-D6 p = 0.004) and nRE (rma: F(2, 24) = 7.048, p < 0.004; ph: D2-D6 p = 0.002), but not the vCA1 (rma: F(2, 20) = 3.263, p = 0.059), suggesting enhanced engagement of mPFC and nRE with enhanced delay (Figure 2B) (see also^15^^,^^38^). However, the degree of phase coherence did not change significantly between regions in this frequency range (Figure 2C).

In the beta range power increased significantly in the mPFC (rma: F(2, 24) = 3.888, p = 0.035; ph: D2-D6 p < 0.0001), showed no significant change in the nRE (rma: F(2,24) = 1.721, p = 0.20) but decreased in vCA1 (rma: F(2, 20) = 4.044, p = 0.034; ph: D2-D4 p = 0.0002, D2-D6 p = 0.0002) (Figure 2B). However, when we tested for interregional coherence in the beta range we found a specific enhancement between the nRE and vCA1 with extended delay periods in the choice phase of the WM task (Figure 2C; rma: interaction F(4, 28) = 5.251, p = 0.003; ph D2-D6 p = 0.009), suggesting that communication between nRE and vCA1 increased during choice with longer delay intervals.

Power in the gamma frequency band was unchanged in the nRE (rma: F(2, 24) = 2.049, p = 0.15), marginally increased in mPFC (rma: F(2, 24) = 2.980, p = 0.07; ph: D2-D6 p = 0.0005) and significantly decreased in vCA1 with prolonged delay intervals (vCA1, rma: interaction F(4, 40) = 4.630, p = 0.004; ph: D2-D4 p < 0.0001, D2-D6 p < 0.0001) (Figure 2B). Remarkably, however, we found significant increases in gamma coherence between both the mPFC and vCA1 (rma: interaction F(4, 28) = 16.02, p < 0.0001; ph: D2-D4 p < 0.0001, D2-D6 p < 0.0001) as well as the nRE and vCA1 (rma: interaction F(4, 28) = 4.166, p = 0.009; ph: D2-D4 p = 0.014, D2-D6 p < 0.0001) (Figure 2C), corroborating our initial findings of enhanced gamma coherence between these structures during WM compared to non-WM tasks. Interestingly, the switch from positive to negative phase shift values in WM vs. non-WM tasks between nRE and vCA1 (Figure S3) was maintained during all delay durations (average phase shift: D2 -45.3° ± 9.5; D4 -40.5° ± 12.3; D6 -25.1° ± 9.2 vs. non-WM task +52.9° ± 20.9).

Since both WM performance as well as interregional coherence changed with the duration of the delay interval, we tested whether changes in interregional coherence correlated with changes in behavioral performance. Interestingly, whereas mPFC-vCA1 coherence was not significantly correlated with performance (r = −0.128, p = 0.55), we found significant negative correlations specifically for nRE-vCA1 coherence in both the beta and gamma frequency range (Figure 2D), relating a high nRE-vCA1 coherence during the choice phase to reduced accuracy. However, since we do not know to which extent this correlation is independent of the delay duration, we cannot conclude a causality.

In summary, these data showed that increased delay intervals induced brain region-specific changes in power with a general trend of enhanced power in mPFC and reduced power in vCA1. In addition, increased WM demands caused a decrease in WM performance and an increase in interregional coherence specifically between mPFC and vCA1 (gamma) as well as nRE and vCA1 (beta and gamma), with the latter showing negative correlation with task performance.

### Coherence between mPFC, nRE and vCA1 during maintenance declines over time

Increasing the difficulty of the WM task caused reduced performance and enhanced oscillatory coherence between mPFC, nRE and vCA1 in the choice phase. As task difficulty was solely determined by delay duration, we reasoned that the cause for any changes in performance and network activity during the choice phase must lie in the preceding delay period (memory maintenance; time between sample touch and illumination of the test locations). Accordingly, we compared network activity between the different delay intervals D2, D4 and D6. Basic levels of power and coherence during maintenance were very similar to those during choice for all frequency ranges we analyzed. Also, the increase in power with delay durations were similar to those during choice (Figure S4).

**Figure 3:**
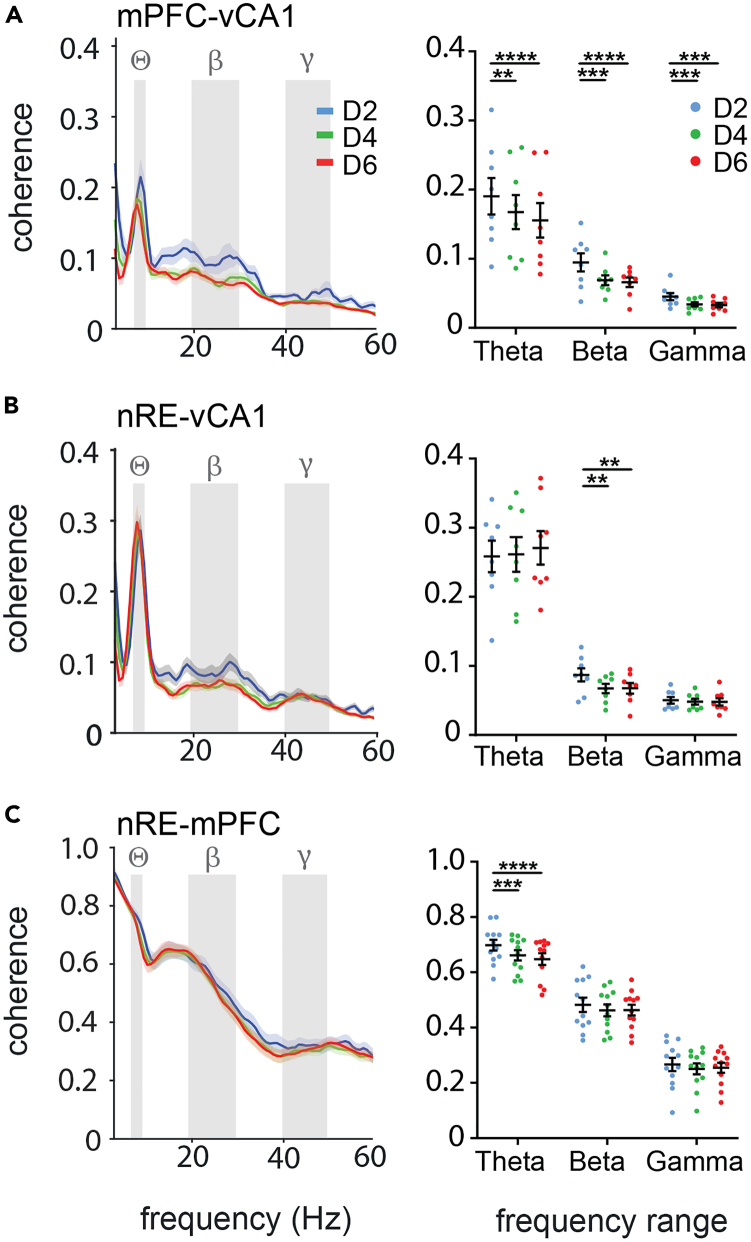
Oscillatory coherence between mPFC, nRE and vCA1 during WM maintenance is lower at long delay intervals (A–C) Spectral (left side) and average (right side) coherence during the maintenance phase for D2 (blue), D4 (green) and D6 (red) sessions for the mPFC-vCA1 (A), nRE-vCA1 (B) and nRE-mPFC (C) connections revealed delay duration-dependent reductions at specific frequencies for different parts of the network. Gray fields indicate core frequencies. Abbreviations: mPFC, medial prefrontal cortex; nRE, nucleus reuniens; vCA1, ventral CA1; Θ, Theta; β, Beta; γ, Gamma. Data are shown as mean ± SEM. ∗∗p ≤ 0.01, ∗∗∗p ≤ 0.001, ∗∗∗∗p ≤ 0.0001.

However, when we analyzed interregional phase coherence for different delay durations, we found marked differences between maintenance and retrieval periods. In particular, phase coherence between mPFC and vCA1 was significantly reduced during longer delay intervals in all frequency bands (mPFC-vCA1: theta, rma: interaction F(4, 28) = 8.205, p = 0.0002; ph D2-D4 p = 0.005, D2-D6 p < 0.0001; beta, rma: interaction F(4, 28) = 8.631, p = 0.0001; ph D2-D4 p = 0.0002, D2-D6 p < 0.0001; gamma, ph: D2-D4 p = 0.0008, D2-D6 p = 0.0003) (Figure 3A). However, reduced phase coherence was also observed between nRE and vCA1 in the beta (rma: interaction F(4, 28) = 8.631, p = 0.0001; ph D2-D4 p = 0.0002, D2-D6 p < 0.0001) as well as mPFC and nRE in the theta range (rma: interaction F(4, 44) = 5.676, p = 0.0009; ph D2-D4 p = 0.0003, D2-D6 p < 0.0001) (Figures 3B and 3C, respectively).

**Figure 4:**
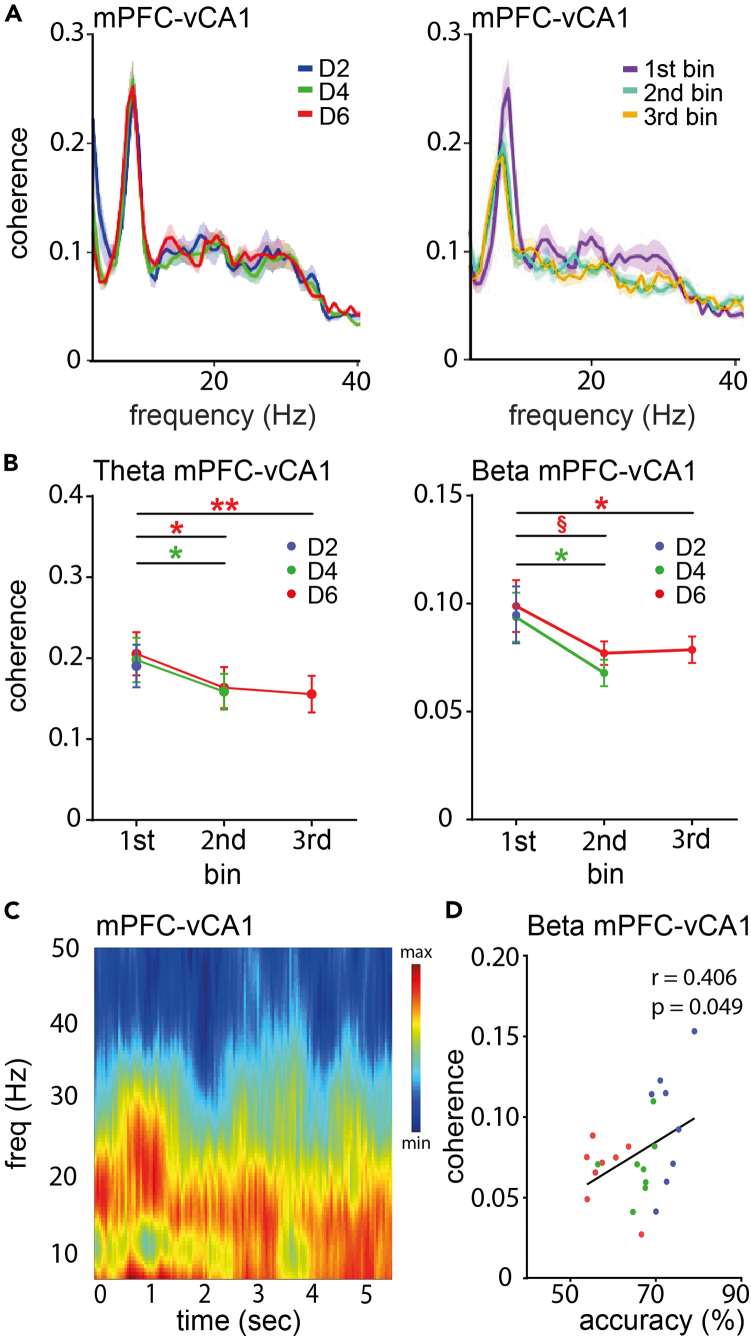
Oscillatory coherence during WM maintenance between mPFC and vCA1 degrades over time and correlates with choice accuracy (A) Left: Coherence between mPFC and vCA1 was similar during the first 2 s of the delay period in D2, D4 and D6 sessions. Right: To test whether coherence deteriorated over time at longer delay intervals, we divided D6 into three 2-s bins. Spectral analysis showed that coherence was strongly reduced in the second and third bin compared to the first. (B) Quantification of oscillatory coherence during delay between mPFC and vCA1 at theta and beta frequency revealed a significant drop between the first and subsequent 2-s bins. (C) Coherogram visualizing the averaged changes in oscillatory coherence over the 6-s delay interval for all animals during the D6 sessions. (D) Coherence between mPFC and vCA1 during the delay period at beta frequency correlated with WM choice accuracy. Same color code as in A and B. Abbreviations: mPFC, medial prefrontal cortex; vCA1, ventral CA1. Data are shown as mean ± SEM. ∗p ≤ 0.05, ∗∗p ≤ 0.01, § 0.05 < p < 0.1.

As the general procedure of the WM task differed only in the duration of the delay period, we hypothesized that the deterioration in coherence seen in D4 and D6 must occur during the extra time of the delay period. Indeed, we found that theta and beta coherences between mPFC and vCA1 during the first 2 s of D4 and D6 did not differ from D2 (theta: D2 0.19 ± 0.026; 1st bin D4 0.20 ± 0.028; 1st bin D6 0.21 ± 0.027; beta: D2 0.10 ± 0.013; 1st bin D4 0.09 ± 0.011; 1st bin D6 0.10 ± 0.012), but then decreased for D4 and D6 in subsequent 2 s bins (theta D4 1st vs. 2nd bin p = 0.016; D6 rma: F(1.5, 10.6) = 14.56, p = 0.002; ph 1st vs. 2nd bin p = 0.021, 1st vs. 3rd bin p = 0.001; beta D4 1st vs. 2nd bin p = 0.011; D6 rma: F(1.3, 9.2) = 6.8, p = 0.02; ph 1st vs. 2nd bin p = 0.062, 1st vs. 3rd bin p = 0.032; Figure 4), whereas gamma coherence did not change (p > 0.05 for all bins). Similarly, theta and beta coherence between mPFC-nRE and nRE-vCA1, respectively, was not different during the first 2 s (mPFC-nRE theta: D2 0.70 ± 0.019; 1st bin D4 0.68 ± 0.019; 1st bin D6 0.69 ± 0.017; beta: D2 0.48 ± 0.026; 1st bin D4 0.47 ± 0.020; 1st bin D6 0.49 ± 0.019; nRE-vCA1 theta: D2 0.26 ± 0.023; 1st bin D4 0.27 ± 0.028; 1st bin D6 0.31 ± 0.029; beta: D2 0.09 ± 0.009; 1st bin D4 0.09 ± 0.009; 1st bin D6 0.11 ± 0.012) but then declined for D4 and D6 in subsequent 2 s bins (mPFC-nRE theta D4 1st vs. 2nd bin p = 0.049; D6 rma: F(1.9, 21.6) = 17.12, p < 0.0001; ph 1st vs. 2nd bin p = 0.012, 1st vs. 3nd bin p = 0.0001; beta D4 1st vs. 2nd bin p = 0.67(ns); D6 rma: F(1.96, 21.6) = 13.06, p = 0.0002; ph 1st vs. 2nd bin p = 0.67(ns), 1st vs. 3rd bin p = 0.0006; nRE-vCA1 theta no significant changes, p > 0.05 for all bins; beta D4 1st vs. 2nd bin p = 0.010; D6 rma: F(1.6, 11.2) = 5.8, p = 0.02; ph 1st vs. 2nd bin p = 0.076, 1st vs. 3rd bin p = 0.051) (Figure S5). Accordingly, communication within the mPFC-CA1-nRE network at theta and beta frequency declined significantly over prolonged delay periods most prominently between mPFC and vCA1.

Interestingly, coherence between mPFC and vCA1 during the delay period positively correlated with WM performance, with animals showing high coherence in the beta range during maintenance performing better in the subsequent choice phase (Figure 4D).

Communication was thus enhanced between mPFC, nRE and vCA1 not only during retrieval but also during maintenance of WM. This interregional coherence, however, deteriorated significantly over time when the delay phase was extended, which in turn coincided with reduced WM performance in the choice phase. Although the correlation of coherence and performance is indirect, it is tempting to speculate that a decline in coherence during maintenance may underlie the drop in subsequent WM performance.

## Discussion

The nRE is a major bidirectional relay for information transfer between PFC and HC. This pathway forms a main route for top-down information flow from mPFC to CA1 and at the same time enables data transmission in the opposite hippocampal-prefrontal direction. As the PFC also receives a direct monosynaptic projection from vCA1 the nRE serves as a connector to complete and complement a partly bidirectional hippocampal-prefrontal-thalamic- loop (see Figure 1C). In accordance with its connectivity, inactivation of the nRE disrupts behaviors dependent on hippocampal-prefrontal communication such as WM.^35^^,^^36^^,^^37^^,^^38^^,^^39^^,^^40^^,^^41^^,^^42^^,^^43^^,^^44^ Here we show that neural dynamics and inter-regional communication within this network change with the WM phase and differentially correlate with behavioral performance.

To probe vCA1-mPFC-nRE network dynamics we have used multi-site LFP recordings in a touchscreen-based WM paradigm, which shares some similarity with visuo-spatial WM tests used in primates.^49^^,^^50^ Compared to traditional T-maze tasks this paradigm was significantly more difficult. Mice required more training, tolerated only short delay periods and achieved lower rates of correct responses. On the other hand, this task was presumably less vulnerable toward confounding motor strategies and thus permitted a good temporal separation of the WM-task into different cognitive processes (encoding, maintenance, retrieval). Although oscillatory coupling as measured by coherence of LFPs provides excellent insights into the dynamics of inter-regional communication, it has limitations.^52^^,^^57^ Without evidence that the firing of nearby neurons is modulated with the LFP, its local origin is not proven.^57^^,^^58^ In addition, the measure of oscillatory coherence between brain regions, which are bidirectionally connected via multiple pathways and receive common input from several un-recorded brain regions^23^ does not permit conclusions about the directionality of interactions or the role of connected but unobserved brain regions. Bearing these limitations in mind we report that despite differences in task design, our LFP recordings revealed strong similarities to dorsal CA1-mPFC network dynamics^14^^,^^15^^,^^17^^,^^55^^,^^59^ as well as vCA1-mPFC network dynamics^17^^,^^51^^,^^59^ reported previously for rodents in DNMTSs in T-mazes or two-lever boxes. Most prominently, we found vCA1-mPFC theta coherence to increase in the WM task compared to a non-WM task. Relating coherence levels to choice accuracy, we found that vCA1-mPFC theta coherence correlated with WM performance^13^^,^^14^^,^^15^^,^^17^^,^^38^ (but see^53^). In addition, vCA1-mPFC gamma coherence, which has previously been implicated in different phases of T-maze DNMTS tasks,^18^^,^^19^^,^^38^^,^^53^ was enhanced during choice. As direct anatomical connectivity between vCA1 and mPFC is one-way we estimated directionality of information flow using phase lag analysis. Calculated phase lags were compatible with the prevailing view, that CA1 leads the mPFC at theta and gamma frequency.^14^^,^^15^^,^^16^^,^^17^^,^^18^^,^^24^^,^^38^ However, due to the complex interconnectivity of vCA1 and mPFC^23^ such analysis has to be treated with caution.^57^ Accordingly, the exact type of information transmitted at these frequencies is not known but both theta and gamma have generally been proposed to serve the transfer of spatial information from HC to mPFC.^2^^,^^18^^,^^24^

Beyond the established changes in CA1-mPFC theta and gamma coherence, our experiments have exposed significant increases in beta coherence between mPFC, vCA1 and nRE during WM. Beta oscillations have originally been associated with sensorimotor processing. As they are prominent throughout the motor system during holding periods but are attenuated during movements and as they occur in sensory areas before and after stimulus onset but disappear during stimulus presentation, beta oscillations have been suggested to serve the maintenance of the current sensorimotor set, or “status quo”.^60^^,^^61^ However, in analogy to a role in sensorimotor processing beta oscillations have recently been recorded within and between brain regions engaged in WM tasks in primates^62^^,^^63^^,^^64^ and mice,^53^^,^^55^ where they may serve the maintenance of a “cognitive set”.^60^^,^^61^ Specifically, beta oscillations have been proposed to serve the top-down control of neural ensembles, by shielding the network from irrelevant information to help the application of task rules.^2^^,^^61^^,^^65^ Interestingly, enhanced beta coherence has been suggested to evolve in thalamo-cortical loops^2^ and has recently been recorded between the mediodorsal thalamus and the mPFC during a T-maze WM task in mice^54^ as well as between nRE and dorsal CA1 during a long-term odor sequence memory task in rats.^66^ Inactivation studies strongly argue for a role of the nRE in mediating not only top-down mPFC to HC executive control but also prefrontal-hippocampal coherence at beta frequency.^67^^,^^68^^,^^69^^,^^70^ Our data suggest that top-down control via the nRE may occur at beta frequency.

Taken together we thus hypothesize that within the mPFC-nRE-vCA1 network investigated here, theta and gamma oscillations may carry task relevant bottom-up spatial information, whereas beta oscillations may rather be related to the executive control over this information and may help to attend to relevant information. Reliable information about the direction of information flow in the different frequency bands would help to better estimate the nature of the information conveyed. However, the dense interconnectivity of the three brain regions and their common input from multiple other brain regions, preclude a meaningful analysis of directionality.^57^ Similarly, we cannot gauge the influence of common input on changes in coherence within the mPFC-nRE-vCA1 network. Such influence has previously been reported for the supramamillary nucleus as well as the cerebellar lobulus simplex.^55^^,^^71^^,^^72^ The question of directionality and the role of common input will thus have to be addressed in future studies possibly by combining LFP and unit recordings with rapid pathway selective interventions.^18^ Regarding the roles of ventral versus dorsal CA1 in hippocampal-prefrontal synchronization, previous studies have reported similar WM-related changes in oscillatory coherence with the mPFC for both regions.^14^^,^^15^^,^^17^^,^^18^^,^^51^^,^^53^^,^^55^^,^^59^ Since the ventral CA1 sends much stronger projections to the mPFC than the dorsal CA1 and since reciprocal connectivity with the nRE is also much higher for ventral than for dorsal CA1,^23^ we propose that vCA1 mediates synchronization of mPFC and dorsal CA1 at different frequencies as also suggested by inactivation experiments.^15^ However, direct connections between dorsal CA1 and mPFC as well as nRE exist and may contribute to interregional coherence.^23^^,^^73^^,^^74^

Besides prominent WM-related beta coherence throughout the mPFC-nRE-vCA1 network our study reports another interesting and previously unappreciated finding: Reduced oscillatory coherence during WM maintenance with extended delay periods, which in turn coincided with a reduction in WM performance in the subsequent choice phase. To our knowledge such distributed oscillatory delay activity and its relevance for WM performance has not previously been reported, possibly due to differences in task designs. This delay activity involved communication between pairs of the three structures and may again serve frequency-specific functions: Whereas theta and gamma oscillations may help to maintain or constantly reactivate neuronal spatial representations, beta oscillations maybe involved in the executive control of these representations to meet the specific task demands.^2^^,^^65^^,^^75^^,^^76^

Considering the above the observed network dynamics could be explained as follows: the prolongation of the delay interval from 2 to 4 and 6 s caused a decline in oscillatory delay coherence between mPFC, nRE, and vCA1 in all three frequencies, which would have impaired the maintenance of task specific spatial representations leading to reduced task performance. In the subsequent choice phase the “loss” of task related representations would have enhanced the exertion of executive power in the futile attempt to make the correct decision (note: enhanced nRE-vCA1 top down attention conveyed by beta coherence would also be compatible with enhanced bottom up gamma coherence to transmit relevant spatial information^2^^,^^75^^,^^77^). This hypothesis is supported by the finding that in the beta range reduced coherence between mPFC and vCA1 during the delay correlated significantly with increased coherence between nRE and vCA1 during the subsequent choice phase (r = −0.851, p = 0.015) and both correlated significantly with behavioral performance (Figures 2 and 4).

In summary and irrespective of content or direction of information flow our findings indicate that inter-area oscillatory coherence between mPFC, nRE and vCA1 at theta, gamma and beta frequency correlates with task performance and dynamically changes throughout the different phases of WM.

### Limitations of the study

The analysis of LFP coherence provides comprehensive insights into the dynamics of inter-regional communication. However, LFPs may be influenced by volume conducted potentials that originate elsewhere. In addition, the measure of oscillatory coherence between brain regions, which are connected via multiple pathways and receive common input from un-recorded brain regions does not permit conclusions about directionality or the influence of unobserved brain regions.

## STAR★Methods

### Key resources table

**Table:** 

REAGENT or RESOURCE	SOURCE	IDENTIFIER
**Antibodies**		

rabbit anti-NeuN	AbCam	Cat# AB177487; RRID:AB_2532109
mouse anti-GFAP-CY3	AbCam	Cat# AB49874; RRID:AB_880203
goat anti-rabbit 488	Thermo Fischer	Cat#A11008; RRID:AB_143165

**Chemicals, peptides, and recombinant proteins**		

Paraformaldehyde	Sigma-Aldrich	Cat# 441244; CAS: 30525-89-4
4% Agar	Roth	Cat# 2266.1
Triton X-100	Fisher BioReagents	Cat# BP151-500; CAS: 9002-93-1
Normal Goat Serum NGS	Capricorn Scientific	Cat# GOA-1A,
Mowiol	Sigma-Aldrich	Cat# 81381; CAS: 9002-89-5
Dapi	Sigma-Aldrich	Cat# 32670; CAS: 28718-90-3
Isoflurane	Baxter	Cat# FDG9623
Buprenorphine, Temgesic	Indivior Europe Ltd. Dublin	PZN: 00345928
Pentobarbital, Narcoren	Boeringer Ingelheim	6088986.00.00

**Deposited data**		

Data and code used for coherogram analysis and running speed analysis	Mendeley Data	https://doi.org/10.17632/v6fz34k2dy.1

**Experimental models: Organisms/strains**		

C57Bl/6J mice	Janvier Labs	RRID: MGI:2670020

**Software and algorithms**		

Matlab 2018	The MathWorks Inc, Massachusetts, USA	RRID:SCR_001622
Python 3.11	https://www.python.org/	RRID:SCR_008394
GraphPad Prism version 9	GraphPad Software, Boston, USA	RRID:SCR_002798
Neuroexplorer version 5	Nex Technologies, Colorado Springs, USA	RRID:SCR_001818
G∗Power - Release 3.1.9.7	https://www.psychologie.hhu.de/arbeitsgruppen/allgemeine-psychologie-und-arbeitspsychologie/gpower	RRID:SCR_013726
ABET II touch software	Campden instruments, Loughborough, UK	Model 89505
DAS-32 Cheetah Data Acquisition Software	Neuralynx Inc, Tucson, USA	Cheetah 5.0 version 5.7.4 https://neuralynx.com/software/cheetah

**Other**		

Epifluorescent microscope	Zeiss	Model Axio Imager M2
Glass slides	Roth	Cat #0656
Vibratome	Leica	Model VT1200S
Triangular conditioning chambers	Campden instruments, Loughborough, UK	Model 80614
Stereotactic frame	David Kopf Instruments, USA	Model 940
Dental Cement	Kulzer	Cat# 64707945
Digital Lynx SX acquisition system	Neuralynx Inc, Tucson, USA	Digital Lynx 4SX acquisition system https://neuralynx.com/hardware/digital-lynx-sx
Electrode interface board	Neuralynx Inc, Tucson, USA	EIB-8; https://neuralynx.com/hardware/eib-8
Stainless-steel electrodes (Ø=0.075mm)	Advent research materials, Oxford, UK	Cat# FE6215

### Resource availability

#### Lead contact

Further information and requests for resources and reagents should be directed to and will be fulfilled by the lead contact, Peer Wulff (p.wulff@physiologie.uni-kiel.de).

#### Materials availability

This study did not generate new unique reagents.

### Experimental model and study participant details

All experiments were performed in accordance with the German law on animal protection and approved by the Animal Care and Ethics Committee of the Christian Albrecht University, Kiel.

#### C57Bl/6J mice (RRID: MGI:2670020)

A total of 25 adult male (n=15) and female (n=10) C57Bl/6J mice (RRID: MGI:2670020) were used in this study, of which 14 were used for LFP analysis (9 male, 5 female). Animals were housed in a temperature-controlled environment with a 12-hour light/dark cycle. Two weeks before the start of the behavioral training, animals were housed individually and food intake was measured daily for a week. Thereafter, mice received approximately 80% of daily food intake, allowing the animals after initial weight loss to stabilize at 80-85% of the starting body weight. Animals selected for surgery were taken off food restriction for at least 4 days before the surgery. During the 2-week recovery period, food was available *ad libitum*.

### Method details

#### Behavioral training

Behavioural training and testing was performed in triangular conditioning chambers (Model 80614, Campden instruments, Loughborough, UK) that were placed in sound- and light attenuating Faraday cubicles. Each chamber contained a house light, tone generator, video camera and LCD touch screen with a liquid reward dispenser in the opposing corner (Figure 1). ABET II touch software (Campden instruments, Loughborough, UK) using custom written protocols was used to control the chambers as well as to record behaviour.

The week before training started, mice were handled and food restricted to reach 80% of their starting body weight. During the entire training course, the animals received one training session of 30 minutes per day. Habituation and pre-training followed published protocols.^78^ Pretraining consisted of several sessions in which the animals were trained to touch an illuminated field of the screen (5 options: 1 illuminated, 4 dark) in order to receive a reward from the dispenser (8μl strawberry milk). Once the mice were doing so, to further improve performance, touching of a non-illuminated field was punished by bright lighting of the chamber, the sounding of a tone for 1 second and a time out period, delaying the start of the next trial by 5 seconds. During DNMTS training, the mouse was placed in the recording chamber and one out of five possible locations was lit. The trial was initiated when the mouse touched the illuminated sample location, which in turn caused the light to tun off. Touching of the sample location was rewarded in 33% of cases by 5μl of strawberry milk from the dispenser to encourage the mice to move away from the touch screen during the delay and thus prevent behavioral/mediating strategies. Upon sample touch, the delay period was initiated, which for initial DNMTS training was set to 2 seconds. After the delay, the sample location plus one new test location was illuminated. Responding to the test (non-match) location by touch was considered a correct choice and resulted in a reward (5μl strawberry milk). An incorrect choice (either the sample light or an unlit field) was punished by bright lighting of the chamber, the sounding of a tone for 1 second and a time out period of 5 seconds. Combinations of locations were randomized during the entire session to make the response location unpredictable. Once animals reached stable threshold performance at the 2 second delay (70% accuracy for 3 consecutive days), the animal proceeded to electrode implantation. After a 2-week post-surgery recovery period a subgroup of 6 animals was first re-habituated to touching the screen while tethered using a task in which each touch of a lit location was rewarded (3 sessions) before proceeding to the 2 second delay task. All other mice were directly retrained on the 2 second delay task until performing above threshold again (4 days above 70% accuracy, usually reached within a week). Consecutively, the delay duration was increased first to 4 seconds (8 to 12 sessions per animal) and finally to 6 seconds (5 to 10 sessions per animal).

#### Surgery

Mice were implanted with 6 single stainless-steel electrodes bilaterally in the mPFC, the nRe and the vCA1 (directed at stratum lacunosum-moleculare) for recordings of local field potentials (LFPs). Anesthesia was induced using 3% isoflurane in O_2_ by inhalation and adjusted between 1.5 and 2% isoflurane throughout surgery. The animals were head fixed in a stereotactic frame (David Kopf Instruments, USA) and body temperature was maintained by a heating mat placed underneath the animal. After exposing the skull, small holes were drilled in the skull and electrodes were implanted bilaterally at fixed coordinates (mPFC 2.2 AP/1.2 LM/ −1.8 DV at a 17° angle; nRE −0.82 AP/ 1.55 LM / −4.5 DV at a 17° angle; vCA1 −3.0 AP/ 3.6 LM / −2.8 DV). A stainless-steel screw was placed on the dura of the hind brain for referencing and grounding. Electrodes were attached to an electrode interface board (Neuralynx Inc, Tucson, USA), which was secured on the skull using dental cement. Mice received an intraperitoneal injection with buprenorphine for post-operative analgesia and were allowed to recover in a warm cage until fully awake. After a recovery time of 2 weeks, the animals were re-trained (see above).

#### Electrophysiology

Local field potentials were recorded using stainless-steel electrodes (Ø=0.075mm; Advent research materials, Oxford, UK) and a Digital Lynx acquisition system (Neuralynx Inc, Tucson, USA) with Cheetah software (Neuralynx Inc, Tucson, USA) at a sample rate of 1.6 kHz, band-pass filtered for 1-200Hz. In order to synchronize the recording to the behavioral performance, the ABETII software was programmed to send TTL signals to the recording system throughout the trials (at trial start, sample location on, correct touch, etc.). After acquisition, data was analyzed using NeuroExplorer (Nex Technologies, Colorado Springs, USA). The power spectral density for individual behavioral epochs was calculated by single-taper Fourier Fast Transform (FFT; 1-80 Hz; 512 frequency values; single taper- Hann; 50% window overlap). Full spectral power (1-80Hz) was transformed logarithmically to dB for spectral examination or by percentage of total power for statistical analysis. Coherence analysis was performed using the “coherence for continuous” function in NeuroExplorer software, in which the FFTs of signals x and y are calculated, followed by their spectral and cross-spectral densities ($P_{XX}=FFT\left(X\right)*Conj\left(FFT\left(X\right)\right)$; $P_{YY}=FFT\left(Y\right)*Conj\left(FFT\left(Y\right)\right)$; $P_{XY}=FFT\left(X\right)*Conj\left(FFT\left(Y\right)\right)$. Here Conj(z) is a complex conjugate of z. $P_{XX}$, $P_{YY}$ and $P_{XY}$ values are averaged across the specified behavioral epochs within each session and coherence values are calculated $abs\left(Mean{\left(P_{XY}\right)}^{2}\right)\;/\;abs\left(\left(Mean\left(P_{XX}\right)*Mean\left(P_{YY}\right)\right)\right)$. Coherence phase values are calculated as the phase of $Mean\left(P_{XY}\right)$. Due to the 1-200 Hz bandpass filter, data recorded at frequencies below 3 Hz were considered unreliable and not taken into analysis. Based on spectral analysis, target peak frequency bands were selected for further analysis (theta peak 7-9 Hz, beta peak 19-30 Hz, gamma peak 40-50 Hz). Subsequent analysis, averaging and statistics were performed using custom written MATLAB scripts.

#### Histology and imaging

At the end of the experiment small electrolytic lesions were produced for post-mortem identification of electrode positions. To this end animals were deeply anesthetized by intraperitoneal injection of Pentobarbital (50 mg per 30 g body weight) and 0.5 mA pulses were applied for 5 seconds to all electrodes. Immediately afterwards, animals were transcardially perfused with phosphate buffered saline (PBS, pH 7.4) for 1 minute followed by 4% paraformaldehyde (PFA) in PBS for 12 minutes (approximately 100ml). Brains were removed, post-fixated in 4% PFA for 4 hours and stored in PBS until further processing. For sectioning, brains were embedded in 4% agar and cut into coronal sections on a Leica VT1200S vibratome (thickness 40μm). Free-floating sections were permeabilized in 0.4% Triton X-100 in PBS for 30 min and blocked in PBS containing 4% normal goat serum (NGS) and 0.2% Triton X-100 for 30 min at room temperature. Primary antibodies (rabbit anti-NeuN, 1:1000, AbCam, and mouse anti-GFAP-CY3 conjugated, 1:750, AbCam) were incubated with sections overnight at 4°C in PBS, 2% NGS, 0.1% Triton X-100. Sections were washed three times for 10 min in PBS, 1% NGS at room temperature and incubated with goat anti-rabbit 488 (1:1000, Thermo Fischer) as secondary antibody for 3 hours at room temperature. Sections were then washed once in PBS containing 1% NGS and twice in PBS alone for 10 min. Sections were mounted onto glass slides (Roth) and cover-slipped using Mowiol (Sigma) containing dapi (1:500, Honeywell Fluka). Sections were imaged on a Zeiss Axio Imager M2 epifluorescent microscope with a 10x objective to identify locations of electrolytic lesions.

### Quantification and statistical analysis

Behavioral data was analyzed per session per animal using ABETII software (Campden instruments, Loughborough, UK). Performance was averaged per animal over all sessions within a specific delay interval (D2, D4, D6) and consecutively averaged over animals per delay interval. Animals that were unable to perform the task to threshold at a 2 second delay interval were taken out of the analysis.

For electrophysiological data, only data from animals that were able to perform the task to threshold at a 2 second delay interval post-surgery was used. Data from correct trials only was filtered and analyzed per animal per session using Neuroexplorer (Nex Technologies, Colorado Springs, USA). Values were imported into MATLAB for further processing and averaging. Electrodes showing poor quality recordings and electrodes outside the target areas were removed from analysis. Since recordings from both hemispheres showed no obvious differences regarding changes in power and coherence during the task, we averaged power values from bilateral electrodes and coherence values from bilateral electrode pairs (e.g. left mPFC-left vCA1, right mPFC-right vCA1) within animal per session. Data then was averaged per animal over all sessions within a specific delay interval (D2, D4, D6) and consecutively averaged over animals per delay interval.

Final group sizes for LPF recordings in the DNMTS task with increased delay durations were mPFC n=13, nRE n=13, vCA1 n=11 (power analysis) and mPFC-vCA1 n=8, nRE-vCA1 n=8, nRE-mPFC n=12 (coherence analysis). Final group sizes of LFP recordings in the subgroup of animals in the WM choice vs must touch task (Figures 1 and S3) were mPFC n=6, nRE n=6, vCA1 n=5 (power analysis) and mPFC-vCA1 n=4, nRE-vCA1 n=3, nRE-mPFC n=6 (coherence analysis).

For correlation analysis of running speed and vCA1 theta power, running speed was determined for the last two seconds before touch in must touch (n=107 trials) and WM-choice phases (n=107 trials) in 6 animals using DeepLabCut^79^ and correlated with vCA1 theta power on a trial by trial basis. The number of trials needed for a moderate correlation (r =0.3)^80^ to reach significance was calculated with G-power^81^ (effect size p=0.3, alpha=0.05, power= 9). The trials that were included in the analysis of running speed and theta power were randomly drawn from the recorded trials.

For statistical analysis we have used GraphPad Prism version 9. Data was tested for normality using a Shapiro-Wilk test and analyzed for significance using 2-way-ANOVA (repeated measures, rma; factors delay duration and trial epoch) with a post-hoc Dunnett’s test (ph) for multiple comparisons or paired-sample T-test as appropriate. For correlation analysis, Pearson's correlation coefficient was calculated and linear regression was performed. A p-value equal to or less than 0.05 was considered significant. Unless stated otherwise, all data are shown as mean ± SEM. In figures bars represent means, bars with error bars refer to means ± SEMs; solid lines represent means, shaded areas represent SEMs; dots represent individual data points.

## Data Availability

• Local field potential data reported in this paper will be shared by the lead contact upon request. • Data and code used for the coherogram analysis and the analysis of running speed were deposited on Mendeley at https://doi.org/10.17632/v6fz34k2dy.1. • Any additional information required to reanalyze the data reported in this paper is available from the lead contact upon request.
