# Supplementary material for: Task-specific oscillatory synchronization of prefrontal cortex, nucleus reuniens, and hippocampus during working memory
Source: iScience. 2023 Aug 3;26(9):107532. doi: 10.1016/j.isci.2023.107532 (PMC10450413; doi:10.1016/j.isci.2023.107532)
Supplement: Document S1. Figures S1–S5 [file mmc1.pdf]

iScience, Volume *26*

Supplemental information

Task-specific oscillatory synchronization of prefrontal cortex, nucleus reuniens, and hippocampus during working memory

Johanne Gertrude de Mooij-van Malsen, Niels Röhrdanz, Anna-Sophia Buschhoff, Thomas Schiffelholz, Torfi Sigurdsson, and Peer Wulff

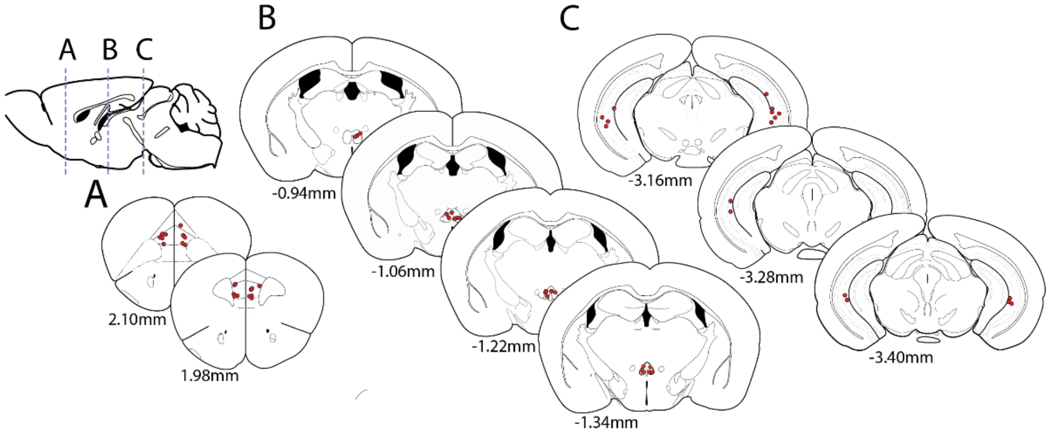

Figure S1. Graphic summary of all electrode positions, related to Figure 1. Rostro-caudally aligned coronal plates illustrating the position of each electrode (red dots) at the level of A) the medial prefrontal cortex, B) the nucleus reuniens and C) the ventral CA1. Numbers below plates indicate anterior-posterior bregma levels. Plates are modified from the Paxinos and Franklin Mouse Brain Atlas.

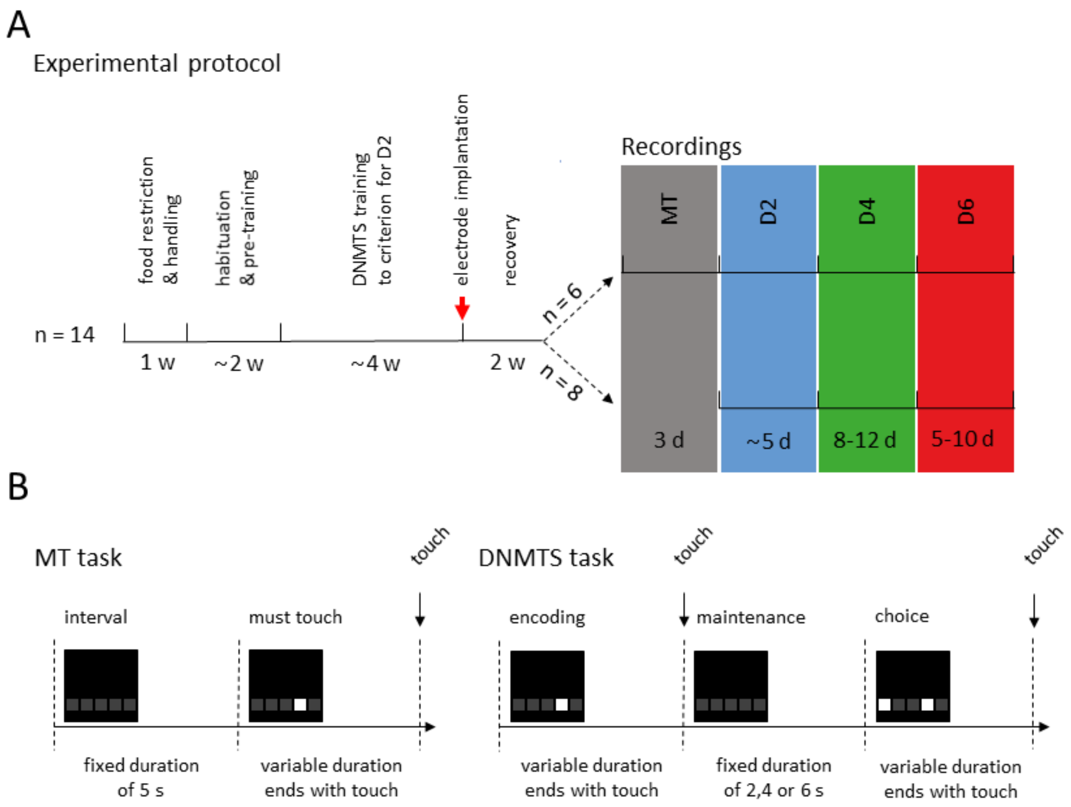

Figure S2. Experimental protocol and time line of non-WM must touch and WM DNMTS tasks, related to Figure 1. A) Illustration of the experimental protocol. All mice used for LFP recordings (n=14) underwent a training protocol for the delayed non-match to sample (DNMTS) task as depicted on the left with the approximate number of weeks (w) needed for each step of the protocol indicated below the time line. After reaching criterion, electrodes were implanted and the animals were allowed to recover for two weeks. For recordings the animals were divided into two groups. One group of six animals started with the non-WM must touch (MT) task before moving into the DNMTS, where they were recorded successively first with a 2 seconds (D2), then 4 seconds (D4) and finally 6 seconds (D6) delay. The other group of eight mice moved straight into the DNMTS task, omitting the MT. The approximate number of days are indicated at the bottom (d, days). Note that each animal only received one session lasting 30 minutes per day both during training and during recording. At the end of the recordings small electrolytic lesions were made and electrode positions were determined histologically. B) Illustration of the time line of the non-WM MT (left side) and DNMTS (right side) tasks. In the MT trial the mouse simply had to touch the illuminated location when it appeared. After the touch a new trial began with the illumination of a single position after a 5 seconds delay. LFPs were analyzed for the last 2s before touch. The sequence of phases for each DNMTS trial was encoding, maintenance with different delays (D2, D4, D6) and choice. The duration of encoding and choice phases were differed from trial to trial as they terminated with the mouse touching the screen. LFPs were analyzed for the entire maintenance phase and the last 2s of the choice phase (just before touch).

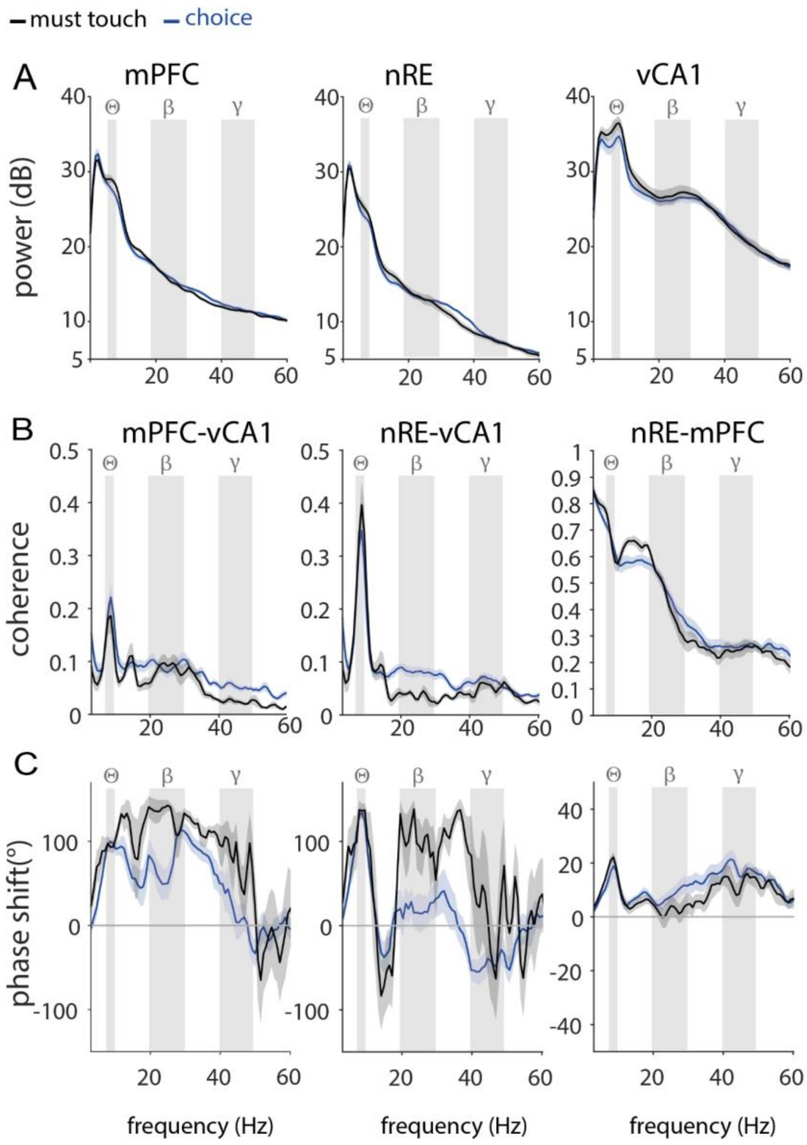

Figure S3. Spectral analysis of local field potentials during non-WM must-touch-task versus working memory choice phase, related to Figure 1. A) Power spectral densities for mPFC, nRE and vCA1 during must-touch (black) versus WM choice phase (blue). Spectral coherence (B) and phase shift (C) analysis for mPFC-vCA1 (left), nRE-vCA1 (middle) and nRE-mPFC (right) oscillatory interactions. Grey fields indicate the core frequency ranges within the theta (Θ), beta (β) and gamma (γ) bands selected for analysis. Abbreviations: mPFC, medial prefrontal cortex; nRE, nucleus reuniens; vCA1, ventral CA1. Data are shown as mean ± S.E.M.

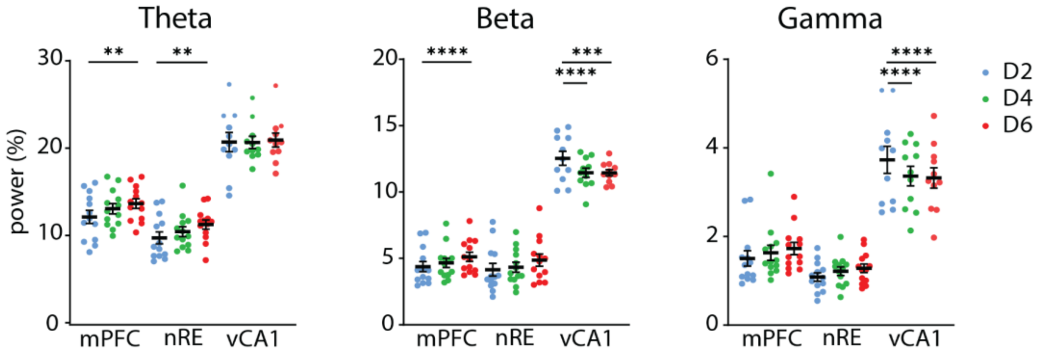

Figure S4. The power of neuronal network oscillations during WM maintenance changes with the duration of the delay interval, related to Figure 3. With longer delay intervals theta power increased in mPFC and nRE, whereas beta power was increased significantly only in mPFC. In contrast, in vCA1oscillatory activity in the beta and gamma range was reduced with longer delay intervals. Shown are mean ± s.e.m. of the percentage of total power during the maintenance phase of D2 (2 s delay, blue), D4 (4 s delay, green) and D6 (6 s delay, red). Abbreviations: mPFC, medial prefrontal cortex; nRE, nucleus reuniens; vCA1, ventral CA1. Data are shown as mean ± S.E.M.. * $p \leq 0.05$, ** $p \leq 0.01$, *** $p \leq 0.001$, **** $p \leq 0.0001$.

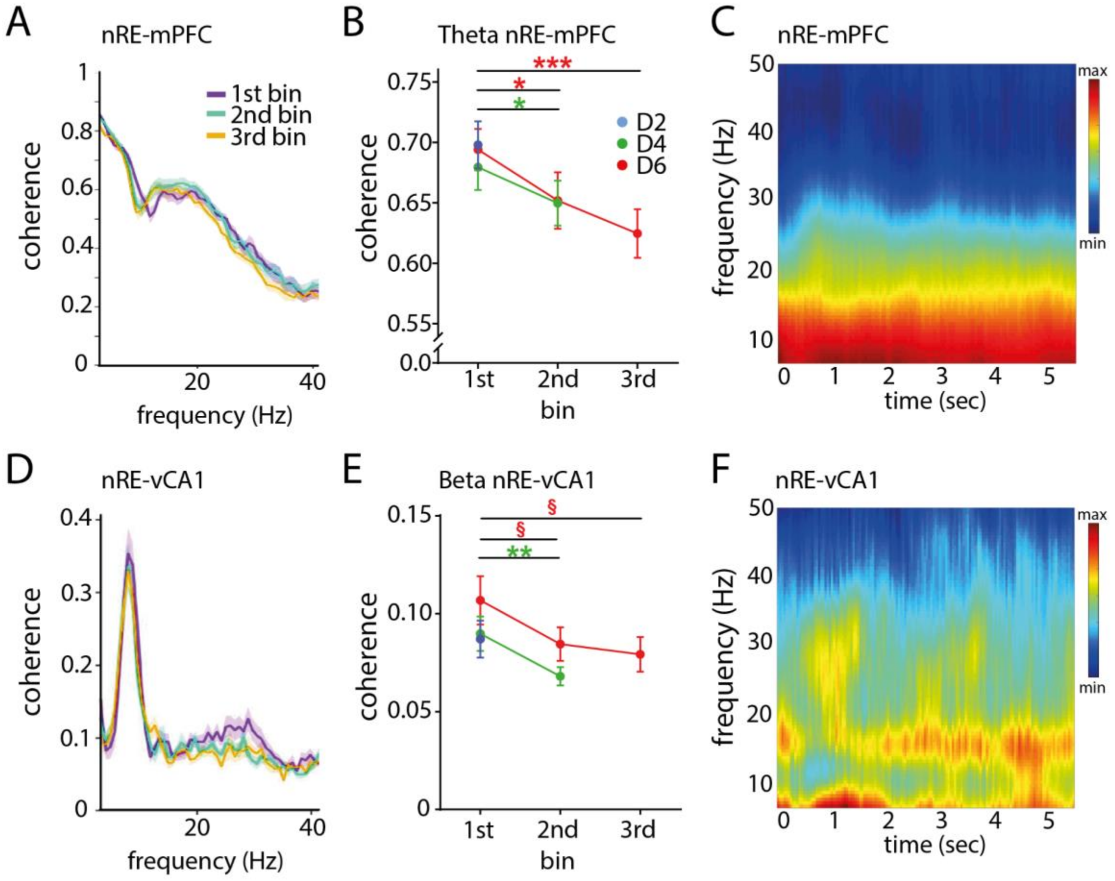

Figure S5. Changes in oscillatory coherence during WM maintenance between nRE and mPFC / vCA1, related to Figure 4. A) Splitting the delay interval into 2 second bins for D6, showed a reduction in theta coherence between the first and subsequent bins between mPFC and nRE. B) Quantification of oscillatory coherence in the theta range during delay revealed a significant drop between the first and subsequent two second bins for D4 as well as D6. C) Coherogram visualizing the changes in oscillatory coherence over time during the 6 second delay period (average of all D6 sessions of all animals). D) Beta oscillatory coherence between nRE and vCA1 during the delay period was higher in the first than in subsequent 2 second bins. E) Quantification of oscillatory coherence in the beta range showed a significant reduction between first and second bin for D4 and a non-significant change between first and subsequent bins for D6. F) Coherogram showing changes in oscillatory coherence over time for D6. (average of all D6 sessions of all animals). Abbreviations: mPFC, medial prefrontal cortex; nRE, nucleus reuniens; vCA1, ventral CA1. Data are shown as mean ± S.E.M.. * $p \leq 0.05$, ** $p \leq 0.01$, *** $p \leq 0.001$, § $0.05 < p < 0.01$.
